# 

**Published:** 1910-12

**Authors:** 


### CONTENTS.

ORIGINAL COMMUNICATIONS—

Treatment of Pulmonary Tuberculosis by Artificial Pneumothorax. By S. T. Harris, of Valdosta, Ga. .................................. 435

Wounds of the Eye. By A. W. Stirling, M. D., M. B., C. M. (Edin.), D. P. H. (Lond.) .................................. 450

Simultaneous Catheterization of the Ureters. By Alfred L. Fowler, M. D., Atlanta, Ga. .................................. 454

Notes on the Mayo Clinic. By R. M. Harbin, M. D., Rome, Ga. .... 459

Can Deaf Mutes be Made to Hear and Talk? By Maury M. Stapler, M. D., Macon, Ga. .................................. 465

EDITORIALS .................................. 471

NEWS AND NOTES .................................. 473

BOOK REVIEWS .................................. 488

